# Sex differences in smoking cessation: a retrospective cohort study in a psychosocial care unit in Brazil

**DOI:** 10.47626/2237-6089-2021-0217

**Published:** 2023-05-02

**Authors:** Bruna Beatriz Sales Guimarães-Pereira, Anderson Sousa Martins-da-Silva, Danielle Ruiz Lima, Carlos Felipe Cavalcanti Carvalho, Aline Rodrigues Loreto, Lucas Pequeno Galvão, Fernanda Piotto Frallonardo, Flavia Ismael, Julio Torales, Antonio Ventriglio, Arthur Guerra de Andrade, João Mauricio Castaldelli-Maia

**Affiliations:** 1 Departamento de Neurociência Faculdade de Medicina Centro Universitário FMABC Santo André SP Brazil Departamento de Neurociência, Faculdade de Medicina, Centro Universitário FMABC, Santo André, SP, Brazil.; 2 Secretaria de Saúde de São Bernardo do Campo São Bernardo do Campo SP Brazil Secretaria de Saúde de São Bernardo do Campo, São Bernardo do Campo, SP, Brazil.; 3 Faculdade de Medicina Universidade Nove de Julho São Paulo SP Brazil Faculdade de Medicina, Universidade Nove de Julho, São Paulo, SP, Brazil.; 4 Departamento de Psiquiatria Faculdade de Medicina Universidade de São Paulo São Paulo Brazil Departamento de Psiquiatria, Faculdade de Medicina, Universidade de São Paulo, São Paulo, Brazil.; 5 Centro de Atenção Psicossocial de Álcool e Drogas São Caetano do Sul SP Brazil Centro de Atenção Psicossocial de Álcool e Drogas (CAPS-AD), São Caetano do Sul, SP, Brazil.; 6 Centro de Estudos em Saúde Mental do ABC Santo André SP Brazil Centro de Estudos em Saúde Mental do ABC, Santo André, SP, Brazil.; 7 Universidade Municipal de São Caetano do Sul São Caetano do Sul SP Brazil Universidade Municipal de São Caetano do Sul (USCS), São Caetano do Sul, SP, Brazil.; 8 Departamento de Psicología Médica Facultad de Ciencias Médicas Universidad Nacional de Asunción Asunción Paraguay Departamento de Psicología Médica, Facultad de Ciencias Médicas, Universidad Nacional de Asunción, Asunción, Paraguay.; 9 Dipartimento di Medicina Clinica e Sperimentale Universitá di Foggia Foggia Italy Dipartimento di Medicina Clinica e Sperimentale, Universitá di Foggia, Foggia, Italy.

**Keywords:** Tobacco use cessation, female, tobacco use disorder

## Abstract

**Introduction:**

Despite the results of epidemiological and psychometric studies reporting comparable levels of tobacco dependence among males and females, some clinical studies have detected disparities. Some smoking cessation studies based on clinical setting programs reported poorer outcomes among women than men.

**Methods:**

This retrospective cohort study aimed to compare treatment success and retention between men and women on a smoking cessation program (n = 1,014) delivered at a CAPS-AD unit in Brazil. The psychological intervention lasted 6 weeks for each group of 15 patients. Each patient had to participate in weekly group cognitive-behavioral therapy (CBT) sessions and individual medical appointments during this period. These appointments were focused on the possibility of prescribing pharmacological treatment (i.e., nicotine replacement therapy, bupropion, or nortriptyline) as adjuvants to group therapy.

**Results:**

The women had lower smoking severity at baseline, more clinical symptoms, and lower prevalence of alcohol and drug use disorders and were older than the men. Females had significantly higher levels of success (36.6% vs. 29.7%) and retention (51.6% vs. 41.4%) than males. Sensitivity analysis showed that female gender was significantly associated with both retention and success, among those without drug use disorders only.

**Conclusion:**

Depending on the smoking cessation setting (i.e., low and middle-income countries and mental health and addiction care units), females can achieve similar and even higher quit rates than males. Previous drug use disorder was an important confounding variable in the gender outcomes analyses. Future studies should try to replicate these positive smoking cessation effects of CBT-based group therapy plus pharmacotherapy in women.

## Introduction

Worldwide, approximately 175 million women are daily smokers, with half of female smokers living in low and middle-income countries.^1^ Due to stigma, females may experience particular difficulties on smoking cessation programs, especially during pregnancy and motherhood.^2^ A large number of factors may affect the outcome, such as caregiving demands, insufficient social backing, hormonal effects, a spouse who smokes, neurobiological variables determining dependence levels, and psychiatric comorbidities.^2,3^

In Brazil, according to the Vigitel/2019 survey (Vigilância de Fatores de Risco e Proteção para Doenças Crônicas por Inquérito Telefônico), 9.8% of adults are smokers, being 12.3% of males and 7.7% of females.^4^ Since 2002, the Brazilian Ministry of Health, in conjunction with state and municipal health departments, has organized a network within SUS (Sistema *Único* de Saúde) healthcare units, aiming to promote and offer smoking treatment. Health professionals administer treatment in individual consultations and support group sessions. Patients who smoke should be helped to understand the role of cigarettes in their lives and be given guidance on how to quit smoking, how to resist the urge to smoke, and, especially, how to live without cigarettes. During the first four group meetings (or individual consultations), support manuals are provided with information on each of the sessions. Pharmacological treatment may also be provided to reduce the symptoms of nicotine withdrawal syndrome.

Despite results of epidemiological and psychometric studies reporting comparable levels of tobacco dependence among males and females,^5,6^ some disparities have been detected in clinical studies.^7,8^ Females more frequently seek treatment than males,^9^ but employ anti-smoking medications less often.^10^ In addition, there are more reports of side effects associated with smoking cessation medication and craving in females.^11-13^ Moreover, females report more concerns about weight gain related to smoking cessation.^14^ According to Cepeda-Benito et al.,^15^ nicotine replacement therapy (NRT), which is a treatment for tobacco dependence that is widely available as worldwide, seems to be less effective in females than in males. Moreover, smoking cessation studies based on programs in clinical settings reported poorer outcomes among women than men.

The literature on this topic presents mixed findings. It was therefore necessary to conduct this study to compare success and retention between males and females participating in a smoking cessation program offered by a Psychosocial Care Center (Centro de Atenção Psicossocial - Álcool e Drogas – CAPS-AD) that treats alcohol and drug problems in the state of São Paulo, Brazil. In the present study, we hypothesized that males would have higher abstinence rates than females at the end of the treatment, as in previous studies with the general population.

This retrospective cohort study aimed to compare treatment success and retention between men and women on a smoking cessation program offered at a CAPS-AD unit in Brazil.

## Methods

### Treatment protocol

Our sample was recruited from people enrolled on a Smoking Cessation Protocol delivered at a CAPS-AD treatment unit located in the city of São Caetano do Sul, in the state of São Paulo, Brazil. São Caetano do Sul has the highest Human Development Index (HDI) in Brazil, according to the Brazilian Institute of Geography and Statistics (IBGE).^16^

The CAPS-AD units are well-known among health professionals and patients seeking addiction treatment (especially alcohol and marijuana) or needing smoking cessation treatment.

As part of the protocol, the unit offers weekly group therapy, including motivational approaches for smoking cessation. Individuals interested in quitting smoking are then evaluated for further inclusion in the smoking cessation treatment.

Patients may join the treatment protocol for smoking cessation in three different ways: 1) being referred by a Primary Care Unit (Unidade Básica de Saúde – UBS); 2) on-demand in a CAPS-AD; and 3) after treatment failure (patients who decide to repeat treatment). People in this category were only included in the present study at the time of their first contact with the smoking cessation treatment.

Patients whose previous treatment has failed (after attending all six treatment appointments with no substance abuse cessation) are invited to join the initial motivational lecture again.

The intervention lasted 6 weeks for each group of 15 patients. During this period, each patient had to participate in weekly group therapy sessions and attend individual appointments with a physician (t1 = 0, t2 = 1 week, t3 = 3 weeks, t4 = 6 weeks).

The topics discussed in the group therapy sessions included risks of smoking, difficulties and benefits of quitting smoking, and relapse prevention. The group-conduction technique is based on the principles of cognitive-behavioral therapy (CBT).

Medical appointments were focused on the possibility of including pharmacological treatment (i.e., nicotine replacement therapy, bupropion, or nortriptyline) as an adjuvant to group therapy. These appointments also evaluated side effects and dose adjustments. The physician could also refer the patient to another specialist or psychiatrist, if necessary, during or after the 6 weeks of treatment. Most of the investigators were psychiatry residents supervised by a qualified preceptor.

### Sample

The participants were interviewed by health professionals (i.e., physicians, psychiatrists, and psychologists) at the service (who were trained by the director of the unit, who is a certified psychiatrist) at four times: t0, t1 (1 week), t2 (3 weeks), and t3 (6 weeks). During this period, weekly group sessions were held. The study sample comprised 1014 patients who completed the smoking cessation program from 2007 to 2016. Patients’ data were collected using a structured questionnaire containing questions on their sociodemographic conditions and smoking habits. The following inclusion and exclusion criteria were applied:

Inclusion criteria:

1. Patients referred by a member of the smoking cessation team at the São Caetano do Sul CAPS-AD unit.
2. Patients who openly agreed to participate in the smoking cessation program.
3. Patients who completed the initial questionnaire.

Exclusion criteria:

1. Patients who did not live in São Caetano do Sul.
2. Patients younger than 18 years and older than 65 years.
3. Patients who did not complete the initial questionnaire.
4. Pregnant women.

### Baseline variables and outcome measures

A preliminary questionnaire was administered and explored 27 variables related to smoking, divided into five groups: sociodemographic, medical, smoking, psychiatric, and environmental profiles.

Patient data were collected with a structured questionnaire, which contained sociodemographic, medical, and smoking profile questions, and then later tabulated. Sociodemographic data collected included: gender, age, education, and income. Medical screening was focused on hypertension, coughing, throat clearing, difficulty breathing, bad physical conditions, paresthesia, palpitation, heartburn, any other symptoms, any other diseases, and being currently on any medical treatment. Smoking information included years of smoking, the number of cigarettes per day, time from waking up until smoking the first cigarette of the day, difficulty avoiding smoking in prohibited places, tendency to smoke even when ill, previous attempts to quit smoking, and the number of other smokers in the patient’s house. The Heaviness of Smoking Index (HSI) was also assessed. This combines the information about time to first cigarette and number of cigarettes per day and measures predictors of cigarette dependence severity.^16^ Surveyed comorbidities included concomitant psychiatric treatment, alcohol-related issues, and problems with any drug. Daily physical activity was also assessed, as well as patients’ supporters in the smoking cessation attempt. The type of pharmacological treatment employed during the treatment, if any, was recorded (i.e., nicotine patch, nicotine gum, bupropion, nortriptyline).

Treatment success was defined as when the patient (i) achieved abstinence of at least 28 days at the end of the treatment period and (ii) completed the 6 weeks of treatment. Both criteria were based on the self-report measures.^17^ In the present study, all patients who did not meet both criteria were considered treatment failures. In addition to treatment success, we also measured treatment retention as a secondary outcome of the present study.

### Statistical analysis

Data were initially organized in a database and later analyzed using STATA version 11 software. The chi-square test was employed for descriptive analysis. All of the 33 variables were then analyzed separately when gender-related. We analyzed the linear correlations of variables with significant differences between genders to include them in the multivariate logistic regression model. Variables with significant correlations were excluded from the logistic regression model based on clinical relevance. Logistic regression models were estimated for successful treatment. Cox survival regression models were estimated for treatment retention. Initially, we employed multivariate survival variable models, including gender and all co-variables. We employed logistic and survival bivariate models including gender and co-variables to investigate possible confounding factors of this co-variable.

### Ethics approval

This study was approved by the Institutional Review Board at the FMABC University Center, with CAAE number 50004314.0.0000.0082.

## Results

**Figure 1:**
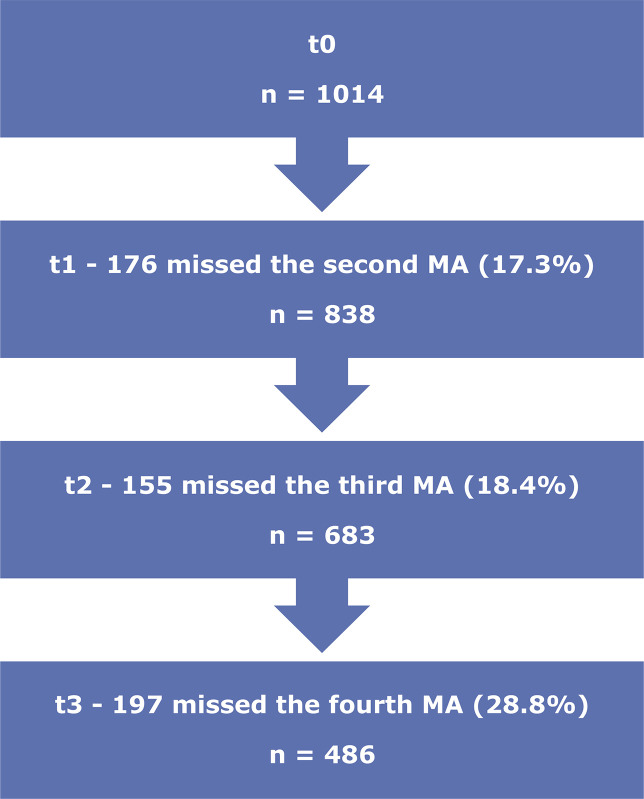
Patient flowchart. MA = medical appointment.

Figure 1 presents the patient flowchart, showing that 1,014 patients started the intervention, 486 attended all 4 medical appointments, and 528 failed to attend all the medical appointments. None of the patients who started the treatment (n = 1,014) were excluded from the analysis in the present study. Sociodemographic data are shown in Table 1. More males had attended undergraduate level education than females (p = 0.022). The most prevalent salary category was from 2 to 3 times the minimum wage for both sexes, but this characteristic was not significantly different. Regarding their medical profile, women reported significantly less phlegm than men (p = 0.022), while men reported significantly fewer symptoms of palpitation (p = 0.004) and significantly less concomitant medical treatment (p = 0.021) than the opposite sex. The other variables did not differ between the sexes (i.e., hypertension, other diseases, coughing, difficulty breathing, decreased physical performance, paresthesia, heartburn, any symptoms).

**Table 1:** Characteristics of a sample of 1,014 smokers treated at a CAPS-AD in São Caetano do Sul, SP, Brazil, 2007-2016

	Female		Male		χ^2^	p

	n	%	n	%		
Totals	654	64.50	360	35.50		
						
Sociodemographic profile						
Age						
Up to 40 years	87	13.45	72	20.34		
41-50 years	178	27.51	75	21.19		
51-60 years	246	38.02	135	38.14		
61 years and older	136	21.02	72	20.34		
Education						
Elementary (partial)	145	22.21	83	23.06		
Elementary (complete)	56	8.58	27	7.50		
High school (partial)	91	13.94	42	11.67		
High school (complete)	181	27.72	80	22.22		
Undergraduate (partial)	75	11.49	72	20.00		
Undergraduate (complete)	81	12.40	40	11.11		
Postgraduate	14	2.14	10	2.78		
None	10	1.53	6	1.67	16.37	0.022
Household income						
Up to 1 times the minimum wage	155	24.33	69	19.55		
2 > 3 times the minimum wage	289	45.37	172	48.73		
4 > 6 times the minimum wage	142	22.29	84	23.80		
7 > 9 times the minimum wage	30	4.71	12	3.40		
10 > 20 times the minimum wage	18	2.83	11	3.12	6.57	0.255
> 20 times the minimum wage	3	0.47	5	1.42		
Medical profile						
Hypertension						
No	470	71.87	265	73.61	0.35	0.551
Yes	184	28.13	95	26.39		
Any disease						
No	427	65.29	242	67.22		0.534
Yes	227	34.71	118	32.78		
Cough						
No	367	56.12	192	53.33	0.72	0.394
Yes	287	43.88	168	46.67		
Phlegm						
No	374	57.19	179	49.72	5.21	0.022
Yes	280	42.81	181	50.28		
Shortness of breath						
No	267	40.83	160	44.44	1.24	0.264
Yes	387	59.17	200	55.56		
Poor physical performance						
No	360	55.05	175	48.61	3.85	0.50
Yes	294	44.95	185	51.39		
Tingling						
No	448	68.50	262	72.78	2.02	0.155
Yes	206	31.50	98	27.22		
Experiencing heart palpitations						
No	490	74.92	298	82.78	8.26	0.004
Yes	164	25.08	62	17.22		
Heartburn						
No	466	71.25	267	74.17	0.98	0.321
Yes	188	28.75	93	25.83		
Any symptoms						
No	54	8.26	26	7.22	0.34	0.321
Yes	600	91.74	334	92.78		
Currently undergoing medical care						
No	221	33.79	149	41.39	7.75	0.021
Yes	433	66.21	210	58.33		
Smoking profile						
Difficulty being in non-smoking areas						
No	273	41.87	173	48.46	4.05	0.044
Yes	379	58.13	184	51.54		
Most difficult cigarette to quit						
Any other	425	66.61	226	63.66	0.88	0.348
First	213	33.39	129	36.34		
Smoke most of the day during sick leave						
No	208	32.60	149	42.57	9.73	0.002
Yes	403	67.40	201	57.43		
Psychiatric profile						
Currently undergoing psychiatric treatment						
No	474	72.48	253	70.28	0.55	0.457
Yes	180	27.52	107	29.72		
Alcohol use disorder						
No	600	91.74	227	63.06	127.04	0.000
Yes	54	8.26	133	36.94		
Drug use disorder						
No	557	85.43	211	58.61	91.16	0.000
Yes	95	14.57	149	41.39		
Environment						
Identified their families as encouraging them to quit						
No	68	10.49	45	12.57	0.99	0.318
Yes	580	89.51	313	87.43		
Any regular physical activity						
No	473	72.32	252	70.00	0.61	0.433
Yes	181	27.68	108	30.00		
Quitting smoking recommended by a doctor						
No	406	62.08	226	62.78	0.04	0.826
Yes	248	37.92	134	37.22		

	**Female**		**Male**			
		
	**Mean**	**SE**	**Mean**	**SE**	**t**	**p**

Profile						
Age	52.50	0.42	50.92	0.62	2.13	0.032
Number of years smoking	32.52	0.42	32.67	0.67	-0.19	0.844
HSI	2.09	0.04	2.33	0.05	-3.63	0.0003
Previous attempts to quit	1.83	0.06	2.03	0.08	-1.81	0.070
Other smokers at home	0.90	0.04	0.81	0.05	1.30	0.192

HSI = Heaviness of Smoking Index; SE = standard error.

Regarding their smoking profile, women reported greater difficulty not smoking in prohibited areas (p = 0.044) or not smoking when ill (p = 0.002) than men. Men had higher prevalence of alcohol use (p > 0.001) and drug use (p > 0.001) disorders than women. Being on treatment for other mental and substance use disorders was not statistically significant between sexes.

Investigation of the environment and lifestyle of the individuals in the sample revealed that more than 85% of both sexes had encouragement from family members to quit. In contrast, minorities reported regular physical activity and medical advice to quit smoking. However, none of the variables analyzed in this category were relevant to the aim of this study. Differences in years of smoking, previous attempts to quit, and presence of other smokers in the house were not statistically significant between sexes. However, the differences in age (p = 0.032) and smoking heaviness index (HSI) (p = 0.0003) were both significantly different between men and women.

Table 2 shows the prevalence rates of types of adjuvant treatment employed: men used more pharmacological aids, nicotine patches (p = 0.086), nicotine gums (p = 0.636), bupropion (p = 0.879), and nortriptyline (p = 0.144) than women. We did not find any significant association between the type of treatment and success rates in either sex (p > 0.05). Females had significantly higher levels of success (36.6% vs. 29.7%, p = 0.027) and retention (51.6% vs. 41.4%, p = 0.002) than males.

**Table 2:** Treatments used by a sample of 1014 smokers treated at an addiction care unit in São Caetano do Sul, SP, Brazil, 2007-2016

Type of treatment	Female		Male		χ^2^	p

	n	%	n	%		
Nicotine patch						
No	50	7.65	39	10.83	2.94	0.086
Yes	604	65.30	321	89.17		
Nicotine gum						
No	556	85.02	310	86.11	0.22	0.636
Yes	98	14.98	50	13.89		
Bupropion						
No	351	53.67	195	54.17	0.02	0.879
Yes	303	46.33	165	45.83		
Nortriptyline						
No	619	94.65	348	96.67	2.13	0.144
Yes	35	5.35	12	3.33		

	**Female**		**Male**		**t**	**p**

	**Mean**	**SE**	**Mean**	**SE**		

Type of treatment						
Nicotine patch	17.88	0.28	18.10	0.40	-0.44	0.656
Nicotine gum	0.51	0.06	0.45	0.06	0.64	0.519
Bupropion	91.45	4.23	89.40	5.64	0.29	0.771
Nortriptyline	1.31	0.23	0.85	0.27	1.22	0.221

SE = standard error.

In Table 3, we report the results of linear correlation among nine variables. The strongest correlations were as follows: educational level and being on medical treatment (-0.10); educational level and age (-0.21); palpitation and throat clearing (0.11); palpitation and being on medical treatment (0.12); being on medical treatment and age (0.22); difficulty in not smoking in prohibited areas and difficulty in not smoking while sick (0.32); difficulty in not smoking in prohibited areas and age (-0.13); and difficulty in not smoking while sick and age (-0.20).

**Table 3:** Linear correlations among 9 variables in 1,014 smokers treated at a CAPS-AD unit in São Caetano do Sul, SP, Brazil, 2007-2016

Variables	Education	Phlegm	Palpitations	Medical care	Non-smoking areas	Sick	Any drugs	Age	HSI
Education	1.00								
Phlegm	-0.02	1.00							
Palpitations	-0.01	0.11*	1.00						
Medical care	-0.10*	-0.04	0.12*	1.00					
Non-smoking areas	0.00	0.07	0.07	-0.01	1.00				
Sick	0.11*	-0.01	0.07	0.01	0.32*	1.00			
Any drugs	0.07	0.00	0.00	0.07	0.01	0.03	1.00		
Age	-0.21*	0.01	-0.02	0.22*	-0.13*	-0.20*	-0.03	1.00	
HSI	0.08	0.04	-0.04	-0.02	0.04	-0.02	0.00	-0.03	1.00

HSI = Heaviness of Smoking Index.

* Significant correlations.

Regarding crude cessation rates, 36.60% of the females and 29.72% of the males achieved cessation. In the univariate regression model, female sex was associated with successful treatment (odds ratio [OR] = 0.73, 95% confidence interval [95%CI] = 0.55-0.96, p = 0.027) although this was not confirmed in the multivariate logistic regression model (OR = 0.76; 95%CI = 0.56-1.02; p = 0.072). Table 4 presents the model with adjustment for several possible confounding variables. The sex-adjusted variables for throat clearing (p = 0.022), palpitation (p = 0.020), concomitant medical treatment (p = 0.020), difficulty not smoking while sick (p = 0.029), difficulty not smoking in prohibited areas (p = 0.030), Heaviness of Smoking Index (p = 0.030), and educational level (p = 0.028) were statistically significant. However, problems with any drug were a confounding factor in this analysis (p = 0.099).

**Table 4:** Results of Logistic Regression for treatment success of 1,014 smokers treated at a CAPS in São Caetano do Sul, SP, Brazil, 2007-2016

Variable	OR	z	95%CI		p
Gender (female as the reference category)	0.73	-2.21	0.55	0.96	0.027
Gender (in bold) adjusted for:					
	**0.72**	**-2.28**	**0.54**	**0.95**	**0.022**
Throat clearing	1.16	1.17	0.89	1.51	0.244
	**0.71**	**-2.33**	**0.54**	**0.94**	**0.020**
Palpitations	0.78	-1.51	0.57	1.07	0.132
	**0.71**	**-2.32**	**0.54**	**0.94**	**0.020**
Any other ongoing medical care	0.88	-0.85	0.67	1.16	0.397
	**0.73**	**-2.18**	**0.55**	**0.96**	**0.029**
Smoking while sick	0.74	-2.09	0.56	0.98	0.036
	**0.73**	**-2.17**	**0.55**	**0.97**	**0.030**
Difficulty staying in non-smoking areas	0.94	-0.44	0.72	1.22	0.663
	**0.78**	**-1.65**	**0.58**	**1.04**	**0.099**
Drug use disorders	0.75	-1.67	0.55	1.05	0.096
	**0.73**	**-2.18**	**0.55**	**0.96**	**0.030**
Heaviness of smoking index (HSI)	1.04	0.64	0.91	1.18	0.525
	**0.73**	**-2.20**	**0.55**	**0.96**	**0.028**
Education					
Elementary (partial)	0.45	-1.51	0.16	1.26	0.131
Elementary (complete)	0.50	-1.24	0.17	1.48	0.215
High-school (partial)	0.32	-2.07	0.11	0.94	0.038
High-school (complete)	0.36	-2.16	0.11	0.90	0.031
Undergraduate (partial)	0.50	-1.88	0.12	1.04	0.060
Undergraduate (complete)	0.36	-1.28	0.17	1.44	0.201
Postgraduate	0.32	-1.27	0.08	1.21	0.094

95%CI = 95% confidence interval; OR = odds ratio.

The survival analysis using multivariate Cox regression (Table 5) showed that female gender (aHR =1.24; 95%CI = 1.02-1.50; p = 0.024) was significantly associated with retention. We performed sensitivity analysis for logistic and survival regression models (Tables S1 and S2, available as online-only supplementary material), splitting the sample into those with and without other drug use disorders. Female gender was significantly associated with both retention (HR = 1.28; 95%CI = 1.02-1.59; p = 0.026) and success (OR = 0.70; 95%CI = 0.50-0.98; p = 0.040), only among those without drug use disorders.

**Table 5:** Multivariate Cox Survival Regression for treatment retention: n = 1,014 smokers treated at a CAPS (São Caetano do Sul, SP, Brazil), 2007-2016

Variable	HR	z	95%CI		p
Gender (female as the reference category)	1.24	2.26	1.02	1.50	0.024
Education					
Elementary (partial)	1.81	1.30	0.73	4.44	0.195
Elementary (complete)	1.77	1.30	0.69	4.52	0.227
High school (partial)	2.26	1.76	0.91	5.61	0.078
High school (complete)	2.36	1.89	0.96	5.78	0.059
Undergraduate (partial)	2.23	1.74	0.90	5.51	0.082
Undergraduate (complete)	1.70	1.14	0.68	4.28	0.253
Postgraduate	2.0	1.39	0.73	5.95	0.165
Palpitations	1.18	1.66	0.96	1.45	0.096
Drug use disorder	1.22	1.93	0.99	1.49	0.054
Difficulty staying in non-smoking areas	1.06	0.75	0.89	1.27	0.451
Heaviness of smoking index (HSI)	0.98	-0.35	0.90	1.07	0.725

The regression was performed using the variable failure as the dependent variable (with retention as the reference category).

95%CI = 95% confidence interval; HR = hazard ratio.

## Discussion

In the present study, women presented lower smoking severity at baseline, more clinical symptoms, and lower prevalence of alcohol and drug use disorders, and were older than men. Before adjusting for variables, women were more successful than men at completing the treatment and at quitting smoking. This finding contradicted our initial hypothesis. In the multivariate regression, however, the association between female gender and treatment success was statistically non-significant. After adjusting for each variable, having previous issues with use of alcohol and other drugs was identified as a confounding factor in the analysis.

According to the literature, women having one or more children increases their odds of cessation compared to similarly aged women with no children.^18^ Moreover, the prevalence of smoking decreases substantially in the last three months of pregnancy.^19^ Other potentially important factors may include hormone variation and menstrual cycle, sex/gender differences in use and effectiveness of smoking cessation medication, and sex/gender differences in use of other tobacco products both prior to and subsequent to quitting smoking.^20^ The results of prospective observational and cross-sectional studies are mixed and demonstrate that bio-psycho-social variation in samples across place and time might determine whether or not women or men are less likely to quit smoking.

Having issues with use of other substances is a relevant indicator of vulnerability to smoke, and it has been associated with greater difficulty with quitting smoking.^21^ Regarding sex differences, literature shows that women with a substance use disorder diagnosis are more likely to be regular smokers when compared to men with the same diagnosis.^22^

According to our experience, women may benefit more than men from a smoking cessation program even though several smoking cessation studies based on programs in clinical settings have described poorer outcomes for women when compared to men.^23,24^ However, surveys in the general population have also suggested that this finding is not generalized to all women, since age could be an influencing variable, as demonstrated by Jarvis et al.^25^ They used data from three major national surveys and showed that women under the age of 50 were more likely to have completely given up smoking than men, while among older age groups, men were more likely to have quit smoking than women. In our study, the mean age of both sexes was over 50 years, but women were nevertheless more successful.

Further studies are needed to understand the maintenance of gender-specific smoking patterns better and to develop more specific smoking cessation efforts for vulnerable smoker subgroups. Nonetheless, individuals with a history of substance use disorder are expected to report more difficulty in remaining abstinent after quitting smoking.^26^ Even after a successful treatment, former smokers with a history of other substance use should receive special follow-up attention regarding risk of relapse related to tobacco and other substances.

For females, early implementation of evidence-based smoking cessation treatment, including NRT (such as transdermal patches, chewing gum, etc.), bupropion, varenicline, and individual or group psychotherapies, is suggested.^27-29^ Lack of medication and/or psychotherapy decreases the chance of successfully quitting smoking.^27-29^ Despite seeking treatment more often,^30^ females are more likely to receive less pharmacological treatments when compared to men.^10^

Employing longitudinal data from the International Tobacco Control Four Country Surveys, conducted in Australia, Canada, the UK, and the US (ITC-4), Smith et al.^31^ examined differences between genders in quitting attempts, reasons for quitting, use of smoking cessation medication, reasons for discontinuing smoking cessation medications, and rates of smoking cessation. There were no sex differences in plans to quit, desire to quit, or quitting attempts. However, quitting success was lower among females who did not use any smoking cessation medication.

Mainly in females, there are promising findings on the use of bupropion, varenicline, contingency management, cognitive-behavioral therapy (CBT), and CBT plus pharmacotherapy (especially bupropion). Castellani et al.^32^ investigated short and long-term gender differences in smoking cessation using varenicline, finding similar treatment success rates between males and females, with females presenting more side effects due to medication than males. Waters et al.^33^ reported that contingency management during the smoking cessation might produce higher self-efficacy in females when compared to males. Loreto et al.^34^ evaluated a short-duration smoking cessation protocol using CBT plus pharmacotherapy, showing that females achieve more success than males, as reported in our study. Collins et al.^35^ and Chatkin et al.^36^ employed CBT plus bupropion and found similar success rates for both sexes. Corroborating these results, a meta-analysis investigating brief tobacco interventions in primary care found no significant gender differences.^37^ Finally, since weight gain is a major concern in this subgroup, any intervention addressing this issue might be promising.^38^

Unfortunately, different authors report that a significant number of smokers receiving smoking cessation treatment relapse in the subsequent weeks and months.^39,40^ According to these authors’ data, around half of the successful quitters in the present study should have relapsed. Generally, relapse rates range from 30 to 80%^39,40^ during the following year. Smith et al.^41^ described 6-month abstinence rates as follows: bupropion = 16.8%; nicotine lozenge = 19.9%; patch = 17.7%; patch plus lozenge = 26.9%; and bupropion plus lozenge = 29.9%.

Limitations of this study may include that we did not perform a population-based study (selection-bias). Almost 15% of the women involved in the study reported any drug use disorder, which may not represent all women who seek treatment for smoking cessation in general services. Also, we did not consider the effect of the menstrual cycle on success rates, with premenstrual symptoms associated with higher relapse rates in the literature among women attempting smoking cessation.^42^

## Conclusion

Our study may contribute more information to the current literature on the topic regarding those factors influencing the success rates of smoking cessation treatment programs in women. Depending on the smoking cessation setting (i.e., low and middle-income countries, mental health, and addiction care units), females could achieve similar and even higher quit rates than males. Previous drug use disorder was an important confounding variable in the gender outcomes analyses. Future studies should try to replicate these positive smoking cessation effects of CBT-based group therapy plus pharmacotherapy in women.
