# Supplementary material for: Sex differences in smoking cessation: a retrospective cohort study in a psychosocial care unit in Brazil
Source: Trends Psychiatry Psychother. 2023 May 2;45:e20210217. doi: 10.47626/2237-6089-2021-0217 (PMC10241528; doi:10.47626/2237-6089-2021-0217)
Supplement: Supplementary file 1 [file 2238-0019-trends-45-e20210217-suppl01.pdf]

http://doi.org/10.47626/2237-6089-2021-0217

Table S1 - Sensitivity Analysis for those with and without other drug use disorders (Logistic Regression for treatment success of 1,014 smokers who had treatment at a CAPS, São Caetano do Sul, SP, Brazil, 2007-2016)

**Variable**	**OR**	**z**	**95%CI**		**p**
Those without other drug use disorders					
Gender	0.70	-2.05	0.50	0.98	0.040
Those with other drug use disorders					
Gender	1.07	0.25	0.60	1.91	0.801

95%CI = 95% confidence interval; OR = odds ratio.

Table S2 - Sensitivity Analysis for those with and without other drug use disorders (Multivariate Cox Survival Regression for treatment retention of 1,014 smokers who had treatment at a CAPS, São Caetano do Sul, SP, Brazil, 2007-2016)

**Variable**	**HR**	**z**	**95%CI**		**p**
Those without other drug use disorders					
Gender	1.28	2.22	1.02	1.59	0.026
Those with other drug use disorders					
Gender	1.07	0.45	0.77	1.50	0.655

95%CI = 95% confidence interval; HR = hazard ratio.
